# 4336 Renal Tubular Complement C9 Deposition is Associated with Renal Tubular Damage and Fibrosis in Lupus Nephritis

**DOI:** 10.1017/cts.2020.424

**Published:** 2020-07-29

**Authors:** Shudan Wang, Ming Wu, Luis Chiriboga, Chaim Putterman, Anna Broder, H. Michael Belmont

**Affiliations:** 1 Albert Einstein College of Medicine; 2 NYU Langone Health; 3 Montefiore / Albert Einstein College of Medicine

## Abstract

OBJECTIVES/GOALS: Tubulointerstitial damage in lupus nephritis (LN) is a strong predictor of progression to chronic kidney disease and end stage renal disease (ESRD). While complement activation mediates glomerular injury, the role of complement in renal tubular damage has not been evaluated. We investigated the association between complement activation and tubulointerstitial fibrosis. METHODS/STUDY POPULATION: Patients with LN were selected randomly between July 2014 - July 2016. Chromogenic immunohistochemistry was performed on formalin-fixed, paraffin-embedded, 4-µm human renal biopsy sections using unconjugated, murine anti-human Complement C9 (Hycult Biotech, clone X197) as a marker of the terminal complement activation. Positive control is C3 glomerulopathy and negative control is normal kidney. Tubular basement membrane C9 staining intensity were analyzed on semiquantitative scale 0 to 3 by a renal pathologist. Interstitial fibrosis/tubular atrophy were categorized into low (0–10%), medium (11–20%), or high (≥21%). Clinical parameters were assessed at time of biopsy and 6 months post biopsy. Bivariate associations were assessed between presence of tubular C9 (C9+) and other covariates. RESULTS/ANTICIPATED RESULTS: Renal biopsies from 30 LN studied, 23 (77%) of which had proliferative LN. There were 24 (80%) women, mean (SD) age 33 (12) years. Positive tubular C9 staining was observed in 7/30 (23%) biopsies. At time of renal biopsy, C9+ patients had significantly higher urine protein, compared to C9- patients: median (IQR) 6.2g (3.3-13.1) vs. 2.4g (1.3-4.6), p<0.01. The differences persisted at 6 months after induction therapy: 1.08g (1.0-8.3) in C9+ vs. 0.68g (0.2-2.1) in C9- patients, p = 0.06. There was no significant difference in creatinine at renal biopsy between the two groups. Tubular C9 deposition was associated with interstitial fibrosis: 49% had severe interstitial fibrosis vs. none in the C9- group, p = <0.01. Higher proportion of C9+ patients had moderate NIH Chronicity index: 42.9% vs 8.7% in the C9- group, p = 0.07. DISCUSSION/SIGNIFICANCE OF IMPACT: Tubular C9 deposition is significantly associated with proteinuria, interstitial fibrosis and increased chronicity which predict progression to ESRD and high mortality. This finding suggests that complement activation in the tubules may be linked to proteinuria and contribute to mechanism in tubulointerstitial damage in LN.

